# Short-Chain Alcohols Upregulate GILZ Gene Expression and Attenuate LPS-Induced Septic Immune Response

**DOI:** 10.3389/fimmu.2020.00053

**Published:** 2020-02-03

**Authors:** Hang Pong Ng, Yubo Wang, Scott Jennings, Steve Nelson, Guoshun Wang

**Affiliations:** ^1^Department of Microbiology, Immunology and Parasitology, Louisiana State University Health Sciences Center, New Orleans, LA, United States; ^2^Department of Medicine, Louisiana State University Health Sciences Center, New Orleans, LA, United States

**Keywords:** ethanol, propanol, isopropanol, anti-inflammation, immunosuppression, GILZ, LPS, septic shock

## Abstract

Alcohol differentially affects human health, depending on the pattern of exposure. Moderate intake provides beneficial mood modulation and an anti-inflammatory effect, while excessive consumption leads to immunosuppression and various alcohol use disorders. The mechanism underlying this bi-phasic action mode of alcohol has not been clearly defined. Our previous publication demonstrated that ethanol, in the absence of glucocorticoids (GCs), induces expression of Glucocorticoid-Induced Leucine Zipper (GILZ), a key molecule that transduces GC anti-inflammatory effect through a non-canonical activation of glucocorticoid receptor (1). Here we report that similar short-chain alcohols, such as ethanol, propanol and isopropanol, share the same property of upregulating GILZ gene expression, and blunt cell inflammatory response *in vitro*. When mice were exposed to these alcohols, GILZ gene expression in immune cells was augmented in a dose-dependent manner. Monocytes and neutrophils were most affected. The short-chain alcohols suppressed host inflammatory response to lipopolysaccharide (LPS) and significantly reduced LPS-induced mortality. Intriguingly, propanol and isopropanol displayed more potent protection than ethanol at the same dose. Inhibition of ethanol metabolism enhanced the ethanol protective effect, suggesting that it is ethanol, not its derivatives or metabolites, that induces immune suppression. Taken together, short-chain alcohols *per se* upregulate GILZ gene expression and provide immune protection against LPS toxicity, suggesting a potential measure to counter LPS septic shock in a resource limited situation.

## Introduction

An alcohol is any organic compound in which a hydroxyl group (−OH) is bound to a carbon atom of an alkyl or substituted alkyl group. In daily life, alcohol usually refers to ethanol, also known as grain alcohol or spirits of wine. Because of its mood modulation property, ethanol is one of the most consumed recreational substances, which often leads to abuse. In medicine, ethanol and its similar short-chain alcohols (propanol and isopropanol) are commonly used as antiseptics and disinfectants. Ethanol has long been known to be anti-inflammatory and immunosuppressive. Moderate ethanol intake is associated with reductions in many adverse health conditions, including coronary artery disease, diabetes, hypertension, congestive heart failure, stroke, arthritis, and dementia (2–4). However, excessive ethanol intake has been clearly linked to organ and tissue damage (4–6) and life-threatening medical disorders (7, 8). Alcohol abuse predisposes individuals to infections by bacteria, fungi, and viruses (4, 9–12) and leads to specific defects in innate and adaptive immunity (7, 13). Acute ethanol exposure, *in vitro* as well as *in vivo*, inhibits the production of pro-inflammatory mediators, including TNF-α, IL-1, IL-6, IL-8, and MCP-1 (14), and enhances the production of anti-inflammatory mediators, such as IL-10 (15). Additionally, acute ethanol exposure reduces lipopolysaccharide (LPS)-induced inflammatory response *in vivo* (16, 17) and protects mice against staphylococcal enterotoxin B (18, 19). Multiple inflammatory networks, including AP-1 and NF-κB, are reported to be involved in alcohol tempering host response to LPS and SEB (20). However, the upstream signaling pathways underlying this alcohol immunosuppressive effect have not been clearly defined.

Sepsis is defined as a life-threatening organ dysfunction caused by a dysregulated host response to infection (21, 22), which frequently manifests an initial hyper-inflammatory phase, reflected by fever, shock, and respiratory failure (23). If patients survive the initial phase and sepsis persists, they enter a phase of immunosuppression (22, 24, 25). Septic shock, a subset of sepsis marked by severe circulatory, cellular, and metabolic abnormalities, is associated with a greater risk of mortality than sepsis alone (21). Septic shock caused by LPS, the major component of the cell wall of Gram-negative bacteria, is a common condition encountered clinically (26). To study the disease process, an animal model often employed is the peritoneal challenge of mice with LPS. Strikingly, there are natural mouse strains that are exceptionally resistant to LPS. For example, SPRET/Ei mice are highly resistant to LPS and Gram-negative bacterial infection (27), while C3H/HeJ and C57BL10/ScCr mouse strains are resistant to LPS, but susceptible to bacterial infection (28). Genetic analyses of both have revealed that the C3H/HeJ and C57BL10/ScCr mice are deficient in Toll-like receptor 4 (TLR4) function. In contrast, the SPRET/Ei mice highly express Glucocorticoid-Induced Leucine Zipper (GILZ), a member of the transforming growth factor-beta (TGF-β)-stimulated clone-22 (TSC22) family (29) from the gene located on the X-chromosome (30). GILZ, ubiquitously expressed, is primarily regulated by glucocorticoid receptor (GR) signaling to transduce glucocorticoid (GC) effects (31–34). GILZ is known to regulate cell apoptosis, proliferation and differentiation, and to modulate host immunity and inflammation (35–39). More evidence suggesting the crucial role of GILZ in LPS resistance comes from mice receiving recombinant cell-permeable GILZ protein. The GILZ protein administration leads to increased resistance to LPS and reduced LPS-induced mortality (40). Moreover, overexpression of GILZ protects mice against lethal septic peritonitis (41). Directly related to the current alcohol study, our and others' research indicated that ethanol activates GR signaling in the absence of GCs (42, 43). This activation is through ethanol interplay with the cytoplasmic GR complex, releasing GR without GC coupling. The bare GR enters the nuclei to activate its downstream responsive genes, including GILZ (1), which contributes to ethanol inflammosuppression and immunosuppression.

In the current study, we hypothesized that if ethanol indeed prompts GR-GILZ signaling non-canonically, other short-chain alcohols should share the same effect. To test this hypothesis, we compared ethanol, propanol and isopropanol in their modulation of GILZ expression and their effect on host protection against LPS septic immune response.

## Materials and Methods

### Reagents

Dexamethasone, mifepristone, fomepizole, and common reagents were purchased from Sigma-Aldrich. Lipopolysaccharide (*E. coli*, serotype O111:B4 L2630) was from List Biological Laboratories (Campbell, CA) or from Sigma-Aldrich. Pure anhydrous ethyl alcohol or ethanol (200 proof/100%, Koptec), propanol (Sigma-Aldrich), and isopropanol (2-propanol, Sigma-Aldrich) were obtained commercially.

### Cell Culture and Treatments

Human Mono-Mac-6 (MM6) cells were cultured in advanced RPMI-1640 (Invitrogen, Carlsbad, CA) supplemented with 10% fetal bovine serum (FBS) (HyClone, Logan, Utah), 2 mM GlutaMax (Gibco), 100 U/ml penicillin, 100 μg/ml streptomycin, 0.25 μg/ml amphotericin B, OPI media supplement, and non-essential amino acids. The cells were incubated in 5% CO_2_ at 37°C. When alcohol and/or LPS were applied, freshly cultured MM6 cells were exposed to ethanol, propanol, or isopropanol at the level of 50 mM for 24 h in a respective alcohol presaturated incubator. LPS (1 μg/ml) was added 1 h after alcohol addition, and kept in the system until cell harvest. Cells were pelleted and supernatants were collected for cytokine measurements.

### Real-Time Quantitative PCR (RT-qPCR) and Immunofluorescent Staining to Measure GILZ Gene Expression in MM6 Cells

#### RT-qPCR of GILZ mRNA

MM6 cells (1.5 × 10^6^) were exposed to one of the short-chain alcohols (ethanol, propanol or isopropanol) at a 50-mM concentration or 1 μM dexamethasone (Dex) for 24 h in the presence or absence of 5 μM mifepristone. The cells were harvested and washed twice with 1x PBS. Total RNAs were extracted using the Qiagen RNeasy Kit. The cDNAs were synthesized using the Quantitect Reverse Transcriptase Kit (Qiagen). Human GILZ primers (GILZ-F: 5′-CATGGAGGTGGCGGTCTATC-3′ and GILZ-R: 5′-CACCTCCTCTCTCACAGCGT-3′) and Glyceraldehyde 3-phosphate dehydrogenase (GAPDH) primers (GAPDH-F: 5′-AAGGTCGGAGTCAACGGATTTGGT-3′ and GAPDH-R: 5′- ACAAAGTGGTCGTTGAGGGCAATG-3′) were used at a final concentration of 500 nM, as published previously (1). The final reaction for each sample was brought to a total volume of 20 μl with RT SYBR green qPCR mastermix (Qiagen). All reactions were carried out in duplicate on a CFX96 system (Bio-Rad Laboratories, Hercules, CA) for quantitative real-time PCR (qPCR) detection. The qPCR data were analyzed by the comparative Ct (ΔΔCT) method. The expression of GILZ of each treated group was compared to that of GAPDH, and normalized to the non-treatment group.

#### Immunofluorescence Staining of GILZ Protein in MM6 Cells

MM6 cells (1.5 × 10^6^) were exposed to ethanol, propanol or isopropanol at a final concentration of 50 mM or dexamethasone (Dex, 1 μM) for 24 h. The cells were then fixed with 4% paraformaldehyde for 1 h at room temperature. The fixed cells were permeabilized with 0.5% Triton X-100/PBS for 1 h, washed with PBS, and blocked with Blocking Buffer [PBS containing 0.1% Triton X-100, 2% donkey serum and 1% bovine serum albumin (BSA)], for 1 h. GILZ expression was detected by staining with a rabbit anti-GILZ antibody (5 μg/ml; Santa Cruz Biotechnology, Dallas, Texas) for 1 h. PE-conjugated F(ab)_2_ donkey anti-rabbit IgG (5 μg/ml; Jackson ImmunoResearch Laboratories, Inc., West Grove, PA) was used as the secondary antibody. The stained cells were analyzed by flow cytometry.

### ELISA Measurements of Human Cytokines From MM6 Cells

MM6 cells were exposed to 50 mM ethanol, propanol, or isopropanol for 24 h. LPS (1 μg/ml) was added 1 h after alcohol addition, and was kept in the system until cell harvest. After centrifuging to pellet cells, the supernatants were collected for TNF-α and IL-6 production measurement using ELISA (R & D Systems, Minneapolis, MN).

### Animal Experiments

This animal research was approved by the LSUHSC Institutional Animal Care and Use Committee (IACUC #3578). Adult C57BL/6 mice (7–12 weeks old, mixed sex) were either purchased from The Jackson Laboratory or produced from our breeding colony.

#### Alcohol Exposure and LPS Challenge

Mice were exposed to ethanol (2 or 4 g/kg), propanol (2 g/kg), isopropanol (2 g/kg) or PBS via intraperitoneal injection. One hour later, a lethal dose of LPS (10 mg/kg, i.p.) was injected. Animals were monitored every 2 h for 36 h post LPS injection. Then, the surviving animals were continuously observed once a day for one more week before termination of the experiment. For experiments where alcohol dehydrogenase inhibitor was applied, fomepizole (10 mg/ml) was injected with the alcohol solutions. For serum cytokine measurements, a separate set of animals that received a similar procedure was reserved. The assigned animals at the indicated time points were anesthetized and bled via cardiac puncture. Serum from each animal was obtained and stored at −20°C until use.

#### Assessment of Alcohol Influence of GILZ Expression in Peripheral Blood Leukocytes

Mice were intraperitoneally injected with dose-escalating ethanol, propanol, or isopropanol at 0, 2, and 4 g/kg, respectively. Peripheral blood was collected 16 h post-injection. After red blood cell lysis, peripheral blood leukocytes were fixed in 4% paraformaldehyde, followed by permeabilization with 0.5% Triton X-100 in PBS for 1 h. After washing with PBS, the cells were blocked with Blocking Buffer (PBS containing 0.1% Triton X-100, 2% donkey serum and 1% BSA) for 1 h. GILZ expression was detected by staining with a rabbit anti-GILZ antibody (5 μg/ml; Santa Cruz Biotechnology, Dallas, Texas) for 1 h. PE-conjugated F(ab)_2_ donkey anti-rabbit IgG (0.25 mg/ml; Jackson ImmunoResearch Laboratories, Inc., West Grove, PA) was used as the secondary antibody. The stained samples were analyzed by flow cytometry.

To examine alcohol influence of GILZ expression in different types of leukocytes, we selected ethanol as the representative alcohol. Mice were administered (i.p.) with PBS or 4 g/kg ethanol for 8 or 16 h. Under CO_2_ anesthesia, blood from each animal was collected via cardiac puncture. After centrifugation, the cell pellet was resuspended in Qiagen red blood cell lysis buffer, and white blood cells (WBCs) were obtained. Next, the WBCs were blocked with TruStain FcTMXPLUS (2.5 μg/ml; BioLegend), and subjected to immunostaining with antibodies against CD11b-FITC (5 μg/ml; Invitrogen), Ly6G-APC (4 μg/ml; BD Pharmingen), CD3e-Alexa 700 (10 μg/ml; BD Pharmingen), CD8-Pacific Blue (5 μg/ml; BioLegend), CD4-PE-Cy5 (10 μg/ml; BD Pharmingen), and CD19-PerCP Cy5.5 (10 μg/ml; BD Pharmingen), Next, the cells were permeabilized and fixed using BD Cytofix/Cytoperm™ Fixation/Permeabilization Kit. Then, the cells were intracellularly stained with GILZ-PE antibody (5 μg/ml; Invitrogen), followed by flow cytometry analysis.

#### Assessment of Phospho-IκB Levels in Peripheral Blood Leukocytes

Adult C57BL/6 mice were exposed to LPS (10 mg/kg) alone or combined with 4 g/kg ethanol for 16 h. Under CO_2_ anesthesia, cardiac puncture was performed to obtain blood. After plasma and cell separation, the cell component was resuspended in Qiagen red blood cell lysis buffer, and the consequent white blood cells were isolated. Next, the cells were blocked with TruStain FcTMXPLUS (0.25 μg/ml; BioLegend), and subjected to immunostaining with the following surface marker antibodies: CD11b-FITC (5 μg/ml; Invitrogen), Ly6G-APC (4 μg/ml; BD Pharmingen), CD3e-Alexa 700 (10 μg/ml; BD Pharmingen), CD8-Pacific Blue (5 μg/ml; BioLegend), CD4-PE-Cy5 (10 μg/ml; BD Pharmingen), and CD19-PerCP Cy5.5 (10 μg/ml; BD Pharmingen). Next, the cells were permeabilized and fixed using the BD Cytofix/Cytoperm™ Fixation/Permeabilization Kit. Then, the cells were intracellularly stained with phospho-IκB-PE antibody (1.25 μg/ml; Invitrogen), followed by flow cytometry analysis.

#### ELISA Measurement of Mouse Cytokines

The collected plasma were diluted appropriately and ELISA was performed to measure the serum level of IL-6, an indicator septic cytokine, using the mouse Duoset ELISA kits (R & D Systems, Minneapolis, MN).

### Statistics

Data were statistically analyzed by Student's *t*-test for differences between two comparing groups. The animal survival data were compared by Log-Rank test. Results were expressed as mean ±SD. Differences with *P*-values smaller than or equal to 0.05 were considered statistically significant.

## Results

### Short-Chain Alcohols Upregulate GILZ Expression in MM6 Cells

**Figure 1:**
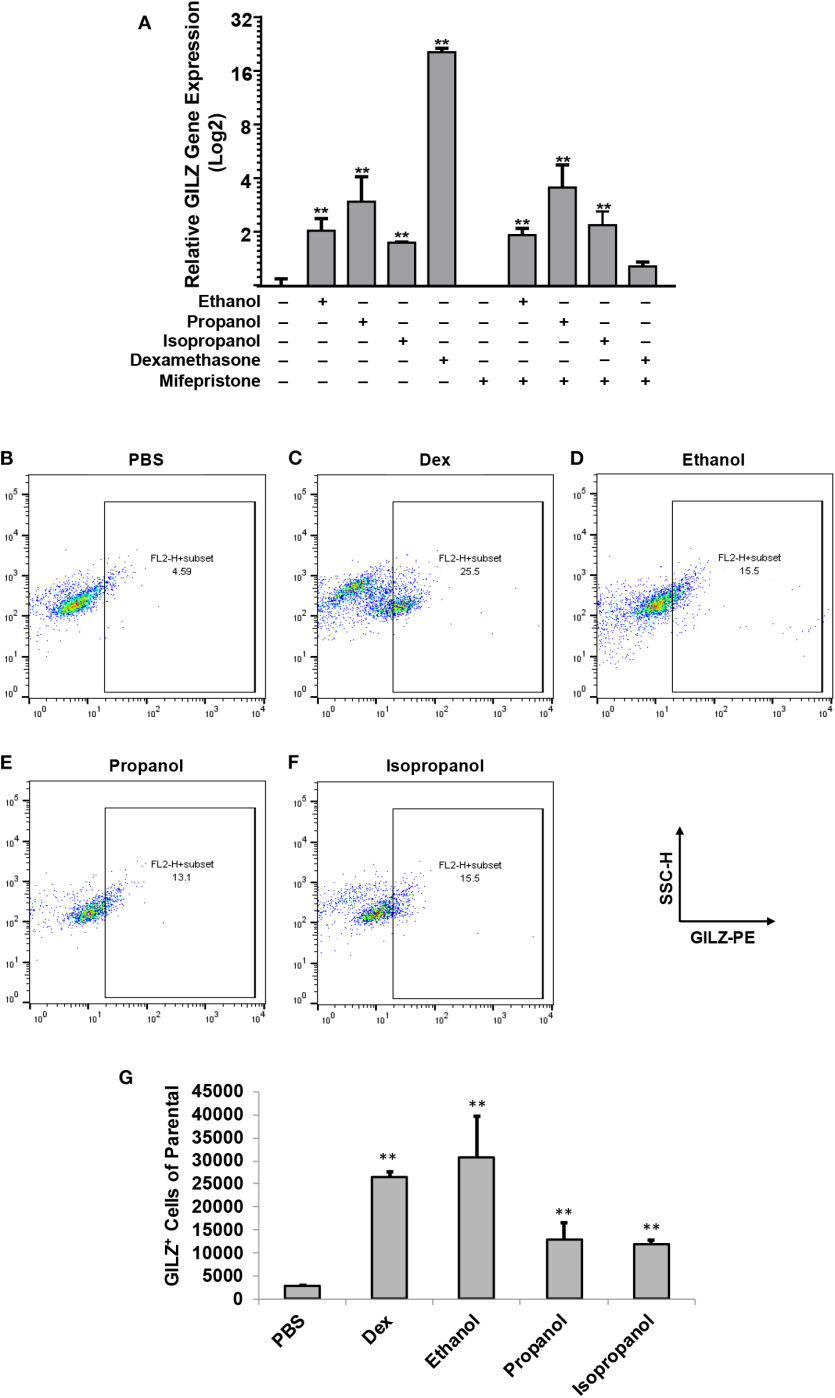
Short-chain alcohols upregulate GILZ expression *in vitro*. **(A)** RT-qPCR to measure GILZ transcription. MM6 cells were exposed to ethanol, propanol, or isopropanol at 50 mM for 24 h. A PBS-control group of MM6 cells was similarly set. GILZ mRNA levels were determined by RT-qPCR. **(B–F)** Flow cytometry. The alcohol-exposed MM6 cells were immunostained for GILZ protein and analyzed by flow cytometry. Representative dot-plot data from each condition are shown. X-axis measures GILZ-PE staining, and Y-axis indicates sidescatter property. **(G)** Statistical data. Total 50,000 cells were acquired for each condition. GILZ-positive cells of the parental were expressed. Asterisks denote significant difference as compared to the respective control by Student's *t*-test (*P* < 0.01, *n* = 3 per condition).

Our previous studies demonstrated that ethanol upregulates GILZ gene expression and suppresses LPS-elicited inflammatory response in human airway epithelial cells and MM6 cells (1, 42). As ethanol, propanol, and isopropanol are all short-chain alcohols with a similar molecular structure, we predicted that they share the same property in regulating GILZ expression and cell inflammatory response. To test this prediction, we exposed MM6 cells to the three alcohols, separately, at 50 mM for 24 h. As GILZ is a glucocorticoid- (GC-) responsive gene, we also stimulated the control group of cells with dexamethasone (Dex, 1 μM). Furthermore, we previously found that ethanol activates the GILZ gene via a GC-independent non-canonical mechanism (1). A parallel experiment was set with addition of mifepristone (5 μM) to block GR. RT-qPCR was performed to measure the GILZ mRNA levels. The results (Figure 1A) demonstrate that the three short-chain alcohols significantly enhanced GILZ gene expression, and mifepristone did little to blunt such an effect. In contrast, the Dex-activated GILZ expression was abolished by mifepristone, suggesting that Dex and the short-chain alcohols exploit different mechanisms to activate *GILZ*. To validate this finding at the protein level, we performed GILZ immunofluorescence staining and flow cytometric analysis. The results (Figures 1B–G) show that ethanol, propanol, and isopropanol significantly elevated GILZ protein expression in the exposed cells, compared to the no alcohol control cells. Taken together, these results indicate that short-chain alcohols are capable of upregulating GILZ expression at both transcriptional and translational levels.

### Short-Chain Alcohols Suppress LPS-Stimulated Inflammatory Response *in vitro*

**Figure 2:**
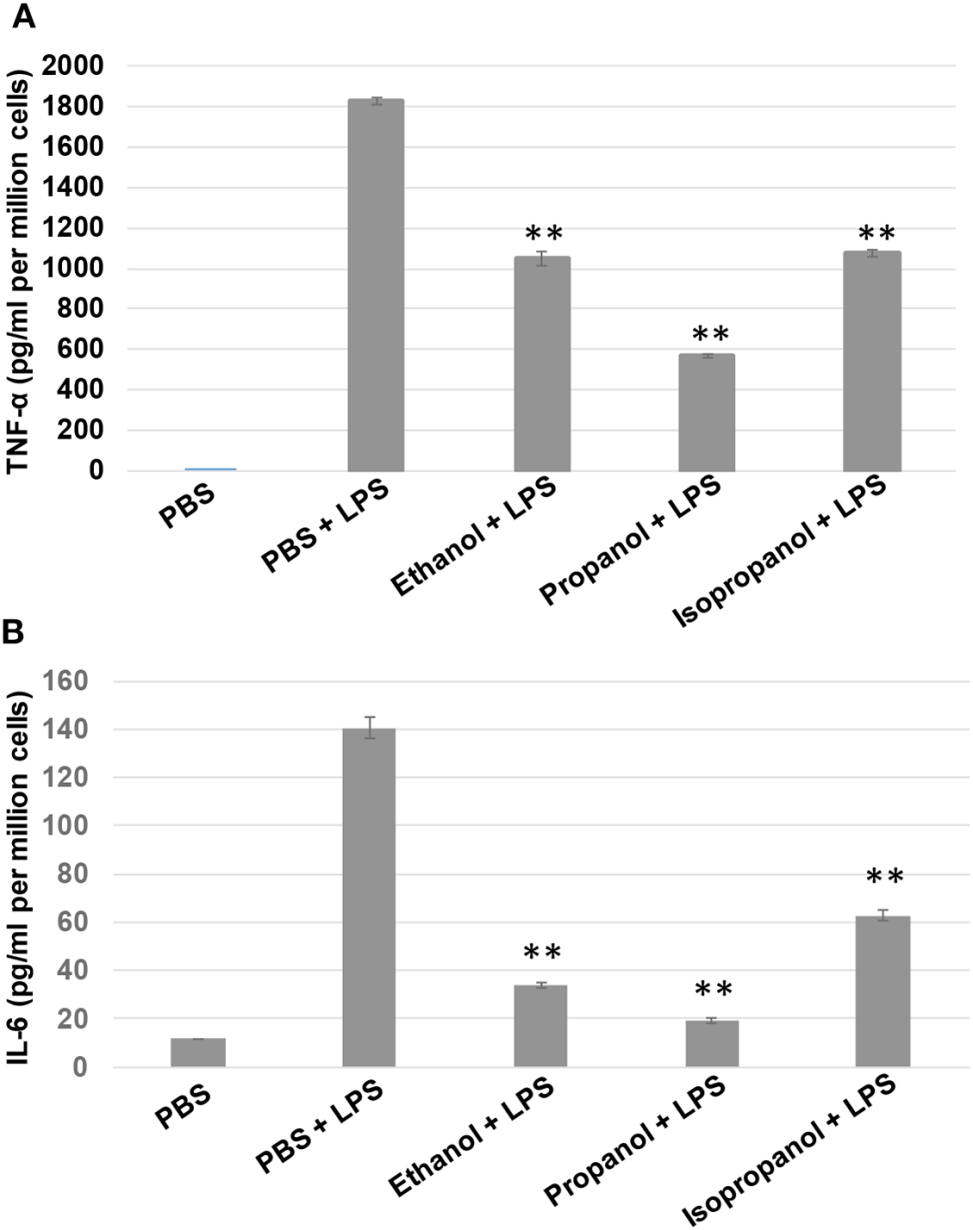
Short-chain alcohols reduce LPS-stimulated TNF-α and IL-6 production by MM6 cells. MM6 cells (1 × 10^6^) were exposed to 50 mM ethanol, propanol, or isopropanol for 24 hours. LPS exposure (1 μg/ml) was added 1 h after the addition of alcohol. A PBS control group of MM6 cells was similarly set and stimulated. Culture media were collected. TNF-α **(A)** and IL-6 **(B)** levels were measured by ELISA, and are expressed here as picogram per milliliter per million cells. Asterisks denote statistically significant difference by student's *t*-test (*p* < 0.01, *n* = 4 per condition).

To examine if propanol and isopropanol behave like ethanol in suppressing cell inflammatory response to LPS, we exposed MM6 cells to 50 mM ethanol, propanol, or isopropanol, followed by LPS (1 μg/ml) stimulation. Levels of TNF-α and IL-6, the two major proinflammatory cytokines in cell response to LPS, in the supernatant of each treatment were determined by ELISA. As shown in Figure 2, the LPS-stimulated cells produced high levels of TNF-α and IL-6, which were significantly reduced by each alcohol.

### Short-Chain Alcohols Enhance GILZ Expression *in vivo*

**Figure 3:**
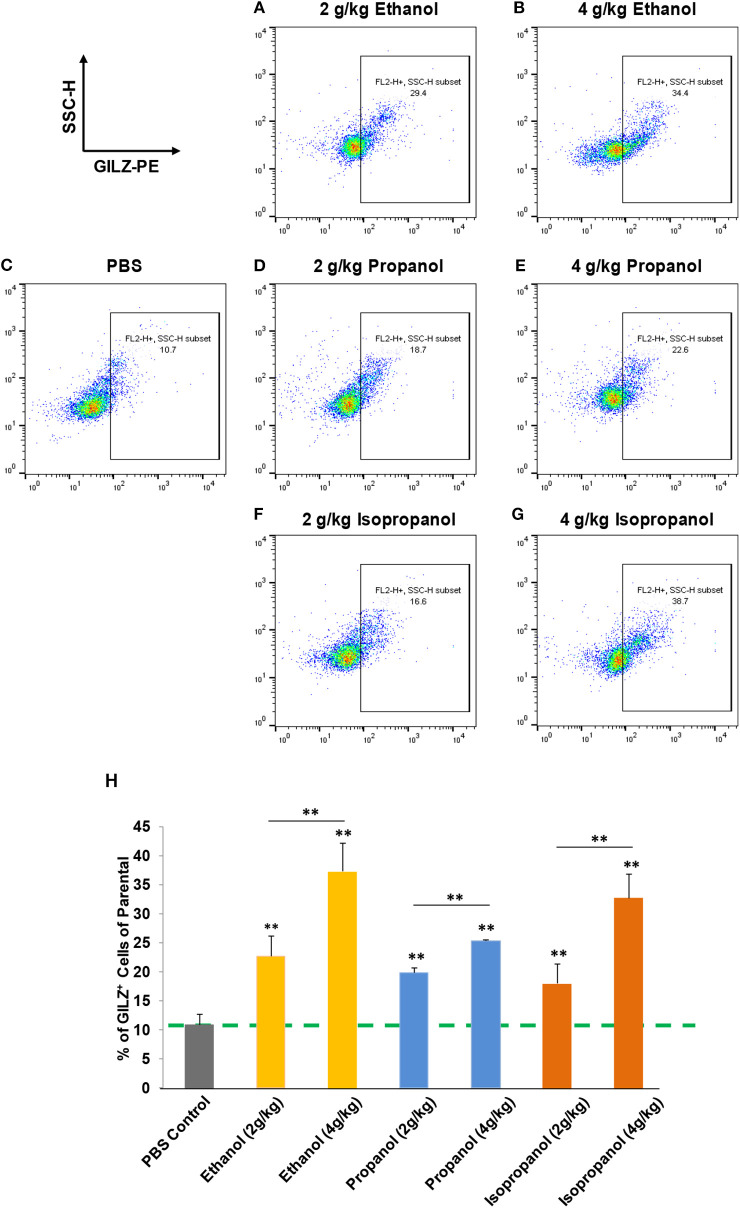
Short-chain alcohols enhance GILZ gene expression *in vivo*. **(A–G)** Dot plot of flow cytometry. Mice were administered (i.p.) with 0, 2, or 4 g/kg of ethanol, propanol, or isopropanol. After 24 h, peripheral blood from each animal was collected, and white blood cells (WBCs) were isolated and subjected to immunostaining for GILZ, followed by flow cytometry. Representative data from each treatment are shown. **(H)** Statistical data. GILZ expression from each condition was compared to that of the PBS control (dashed line). Data are expressed by percent of GILZ-positive cells in each sample. Asterisks denote statistically significant difference in each comparison by Student's *t*-test (*p* < 0.01, *n* = 4 per group).

Short-chain alcohols (ethanol, propanol, or isopropanol) at an escalating dose for each (0, 2, or 4 g/kg) were intraperitoneally administered to adult C57BL/6 mice. Sixteen hours later, white blood cells (WBCs) from each animal were isolated, intracellularly stained for GILZ, and analyzed by flow cytometry. The data (Figures 3A–H) demonstrate that GILZ expression in the cells responded to each of the applied alcohols in a dose-dependent manner, and was significantly higher than that of the non-alcohol treated control.

**Figure 4:**
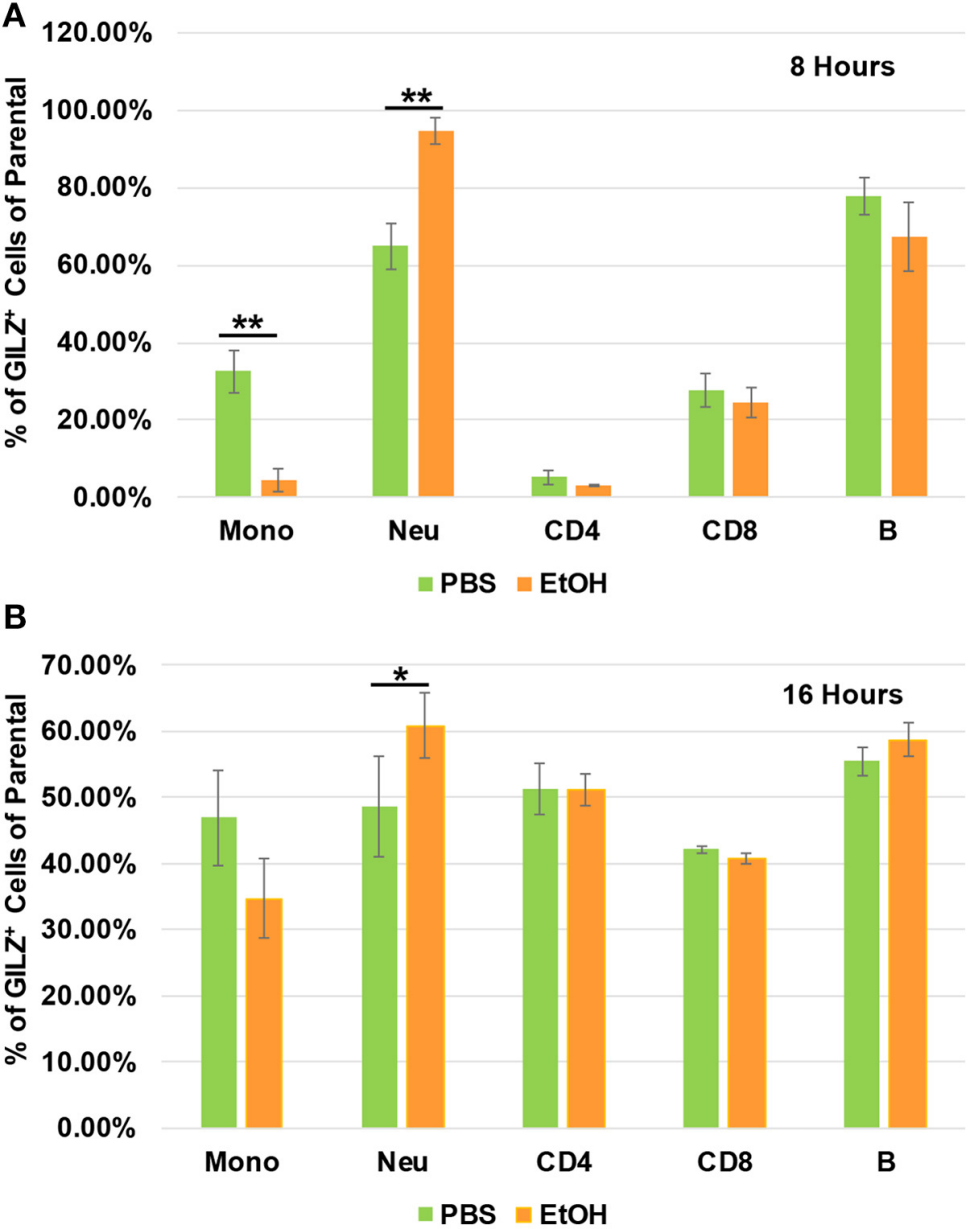
GILZ expression in different types of immune cells. Peripheral blood WBCs from mice that had been exposed to 4 g/kg ethanol or PBS for 8 h **(A)** or 16 h **(B)** were isolated. Immunostainings for cell surface markers (CD11b, Ly6G, CD19, CD3, CD4, and CD8) and GILZ were performed. Monocytes (CD11b^+^Ly6G^−^), neutrophils (CD11b^+^Ly6G^+^), B lymphocytes (CD19^+^), CD4 lymphocytes (CD3^+^CD4^+^), and CD8 lymphocytes (CD3^+^CD8^+^) were categorized. GILZ-positive cells in each cell type of the parental were compared between the ethanol and control groups. Asterisks indicate statistically significant difference by student's *t*-test (*n* ≥ 3 per group). Mono (Monocytes); Neu (Neutrophils). *P ≤ 0.05, and **P ≤ 0.01.

To determine which types of immune cells were altered by alcohol in GILZ expression, we similarly exposed a separate set of mice to ethanol (4 g/kg), a representative alcohol, for 8 or 16 h. Cell-surface staining with antibodies against CD11b, Ly6G, CD19, CD3, CD4, and CD8, in combination with intracellular staining of GILZ was performed. Flow cytometry using the gating strategy (Figure S1) revealed that GILZ expression in monocytes (CD11b^+^Ly6G^−^) was significantly reduced after ethanol exposure for 8 h. However, neutrophils (CD11b^+^Ly6G^+^) had significantly higher GILZ expression (Figure 4A). Moreover, 16 h ethanol exposure led to significantly higher expression of GILZ in neutrophils (Figure 4B). These data suggest that neutrophils are a major cell type in alcohol upregulation of GILZ expression in the current experimental setting.

### Short-Chain Alcohols Protect Mice From LPS Septic Shock

**Figure 5:**
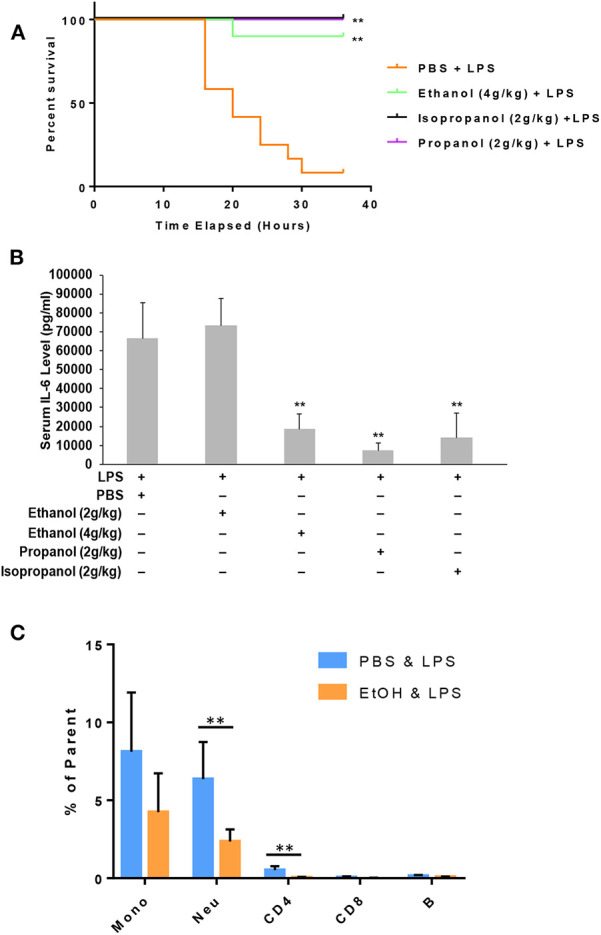
Short-chain alcohol protection against LPS septic shock. **(A)** Survival curves. C57BL/6 mice were i.p. injected with ethanol (4 g/kg), isopropanol (2 g/kg), propanol (2 g/kg), or PBS. One hour later, the animals were challenged with a lethal dose of LPS (10 mg/kg). The animals were observed for 36 h. The Kaplan-Meier survival curve for each group of animals was traced, and statistical comparisons were performed by Log-rank test. Asterisks denote significant difference in each comparison with PBS group or indicated group (*n* = 10 per group, *p* < 0.01). **(B)** Short-chain alcohols attenuate host inflammatory response to LPS. C57BL/6 mice were i.p. injected with ethanol (2 g/kg or 4 g/kg), isopropanol (2 g/kg), propanol (2 g/kg), or PBS. One hour later, the animals were challenged with LPS (10 mg/kg). Sixteen hours after LPS challenge, the animals were bled. Serum from each animal was obtained and measured for IL-6 levels. Asterisks denote significant difference in each comparison (*n* = 4 per group, *p* < 0.01). **(C)** Ethanol exposure significantly affects cellular Phospho-IκB levels in monocytes and neutrophils. Peripheral blood WBCs were isolated from the mice that had been exposed to 4 g/kg ethanol and LPS (10 mg/kg) or the control mice that had been exposed to PBS and LPS (10 mg/kg). Immunostainings for cell surface markers (CD11b, Ly6G, CD19, CD3, CD4, and CD8) and phospho-IκB were performed. Monocytes (CD11b^+^Ly6G^−^), neutrophils (CD11b^+^Ly6G^+^), B lymphocytes (CD19^+^), CD4 lymphocytes (CD3^+^CD4^+^), and CD8 lymphocytes (CD3^+^CD8^+^) were categorized. Percentage of the phospho-IκB-positive cells in each cell type of the gated population were compared between the ethanol and control groups. Asterisks denote statistically significant difference by student's *t*-test (*p* < 0.01, *n* ≥ 3 per group).

Administration of a lethal dose of LPS elicits an overwhelming inflammatory response that leads to multiple organ failure, shock, and death. As short-chain alcohols effectively suppress inflammatory response to LPS *in vitro*, we predicted that they should attenuate LPS-induced septic shock *in vivo*. To test this hypothesis, we administered adult C57BL/7 mice with ethanol (4 g/kg), propanol (2 g/kg), isopropanol (2 g/kg), or PBS control. The reason for selection of a higher dose of ethanol is that our pilot experiments indicated that ethanol at a 2 g/kg dose provided little protection against lethal LPS. One hour after alcohol exposure, the animals were challenged with a lethal dose of LPS (10 mg/kg). A survival curve for each condition was traced, and compared with that of the non-alcohol control (Figure 5A). The results show that without alcohol administration, almost all animals died within the time frame of 16–36 h after LPS challenge, while the short-chain alcohol exposures significantly protected the mice from LPS-induced septic shock. No further casualties were observed after 36 h until termination of the experiment a week later.

LPS-induced septic shock in mice is a well-studied and widely used model, which mimics almost all the pathological consequences that occur during sepsis (44). Mortality caused by sepsis or septic shock is associated with overproduction of inflammatory cytokines, also referred to as cytokine storm (45). IL-6, IL-1β and TNF-α are among the major cytokines responsible for sepsis disease pathogenesis. To delineate the potential mechanism underlying the alcohol LPS protection, we measured the serum level of IL-6, a representative cytokine to indicate the severity of sepsis. Adult mice were exposed to the short-chain alcohols similarly, as previously stated, and challenged with the lethal dose of LPS. Sixteen hours post LPS challenge, all live animals were bled to collect their sera for IL-6 cytokine measurement. Data (Figure 5B) demonstrate that ethanol (4 g/kg), propanol (2 g/kg) and isopropanol (2 g/kg) significantly reduced IL-6 serum levels. However, ethanol at the 2 g/kg dose had no significant impact on LPS-induced inflammation.

One crucial mechanism for ethanol to attenuate LPS toxicity is through suppression of NF-κB signaling (46, 47). To investigate whether this mechanism was involved in the observed alcohol-protection against LPS in our experimental model, we examined the levels of IκB phosphorylation in WBCs from the animals that had been exposed to ethanol (4 g/kg) and LPS. Cell surface marker and phospho-IκB double-immunostaining was performed, followed by flow cytometry analysis using the gating strategy (Figure S2). As compared to the non-alcohol treated cohort, neutrophils and CD4 cells from the alcohol-treated animals had significantly lower phospho-IκB levels (Figure 5C), suggesting that the observed alcohol protection against LPS septic shock is, at least partially, through the suppression of NF-κB signaling.

### Ethanol Instead of Its Derivatives Confers LPS Protection

**Figure 6:**
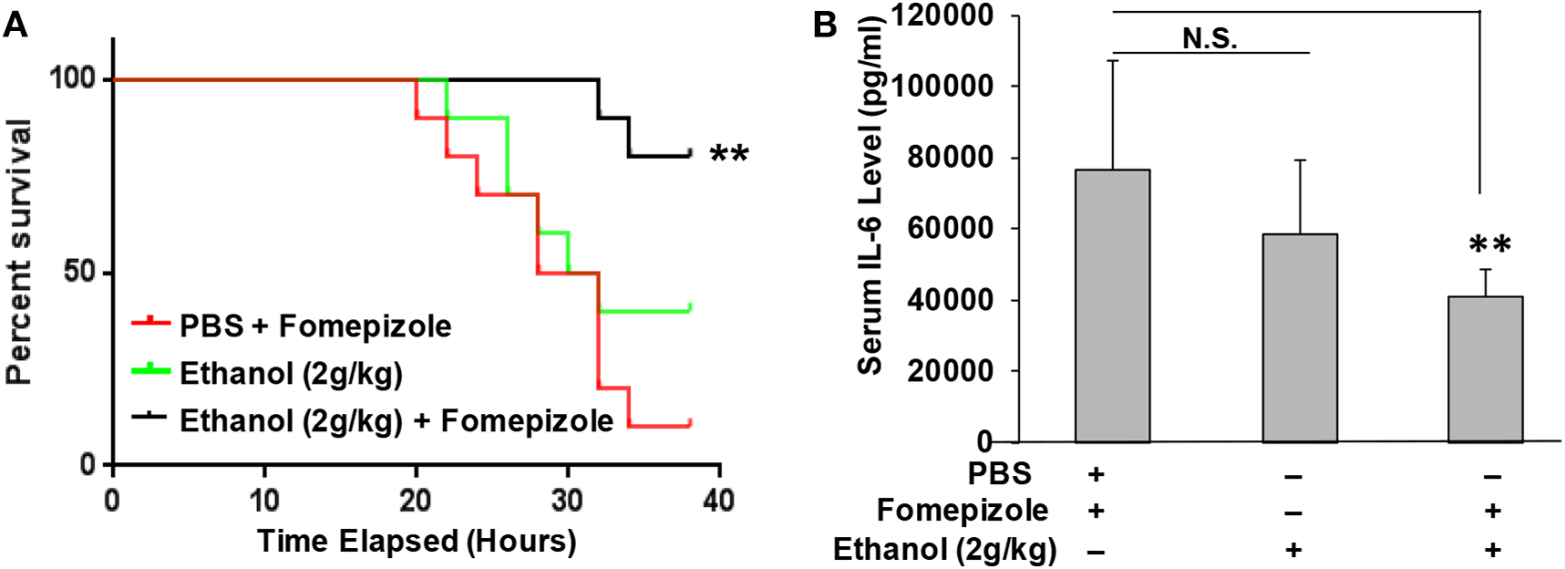
Alcohol dehydrogenase inhibitor fomepizole enhances ethanol suppression of host inflammatory response to LPS and reduces LPS septic shock mortality. **(A)** C57BL/6 mice were i.p. injected with ethanol (2 g/kg) with or without fomepizole (10 mg/kg). One hour later, a lethal dose of LPS (10 mg/kg) was used to challenge the animals. Control mice were injected with PBS and fomepizole. The Kaplan-Meier survival curve for each group of animals was traced, and statistical comparisons were performed by Log-rank test. Asterisks denote significant difference in each comparison (*n* = 10 per group, *p* < 0.01). **(B)** Serum IL-6 levels of the mice with similar ethanol and LPS applications in the absence or presence of fomepizole. Asterisks denote significant difference in the comparison (*n* = 4 per group, *p* < 0.01). N.S. indicates no significance.

It was unexpected that ethanol at the 2 g/kg dose failed to suppress LPS inflammation and protect mice from lethal LPS challenge, while the same dose of propanol and isopropanol were effective. We hypothesized that this may result from a greater or faster metabolic rate of ethanol. If this hypothesis is correct, inhibition of ethanol metabolism may enhance ethanol LPS protection. Three groups of adult C57BL/6 mice were assigned, with one group administered with PBS and the alcohol dehydrogenase inhibitor fomepizole (10 mg/kg), another administered with ethanol (2 g/kg) alone, and the third group with ethanol (2 g/kg) and fomepizole (10 mg/kg) together. One hour later, all animals were challenged with the lethal dose of LPS (10 mg/kg). A Kaplan-Meier survival curve for each condition was traced (Figure 6A) and statistically compared by Log-Rank test. The results demonstrate that without fomepizole, 2 g/kg ethanol had no protective effect on LPS septic shock. However, fomepizole significantly improved the protection efficacy of ethanol at the otherwise non-protective concentration. To further investigate whether fomepizole enhances ethanol suppression of host inflammatory response to LPS, we performed a parallel experiment with the same design. Sixteen hours later after LPS challenge, the serum obtained from each animal was measured to determine the indicator cytokine IL-6 level. As displayed in Figure 6B, fomepizole significantly reduced the serum IL-6 levels, indicating enhancement of ethanol suppression of the host inflammatory response to LPS. Taken together, the data indicate that inhibiting ethanol metabolism facilitates ethanol protection against LPS septic shock, strongly suggesting that it is the molecular ethanol instead of its derivatives or metabolites that engenders the protective effect against LPS-induced septic immune response.

## Discussions

The legal blood ethanol concentration limit for driving in the United States is 0.08%, which is equivalent to 17.36 mM. However, blood ethanol levels can reach to over 87 mM in patients with acute alcohol intoxication (48, 49). A previous study from our group (50) documented that acute alcohol intoxication in mice by intraperitoneal (i.p.) injection of 20% alcohol in pyrogen-free saline at a dose of 5 g/kg gave rise to blood alcohol levels of 119.7 ± 1.3, 106.3 ± 1.5, 87.7 ± 3.6, and 48.4 ± 3.5 mM, respectively, at 45 min, 90 min, 3 h, and 6 h post alcohol administration. In the current study, the highest dose of ethanol used *in vivo* was 4 g/kg. Thus, the blood ethanol level should be lower than the levels referenced. Isopropanol is widely used in household applications. Deliberate or accidental ingestion of isopropanol ranks second as a cause of alcohol poisoning clinically (51). Blood isopropanol concentrations have been reported as high as 560 mg/dl (93 mM) (52). In the current paper, we used 2 g/kg isopropanol for i.p. administration. A previous publication reported that injection of mice with 2 g/kg isopropanol generates a blood alcohol concentration of 200 mg/dl (33 mM) after 30 min (19). Thus, the alcohol doses used in this study should be relevant to clinically encounterable alcohol levels.

Glucocorticoids (GCs) are steroid hormones produced by the adrenal cortex under control of the hypothalamic–pituitary–adrenal (HPA) axis in response to internal circadian clock and external stress challenge (53, 54). GCs are the most prescribed anti-inflammatory drugs. The profound effectiveness of GCs provides the rationale for their use in a wide range of autoimmune, inflammatory, and allergic diseases, such as rheumatoid arthritis, lupus erythematosus, inflammatory bowel disease, transplant rejection and asthma (55, 56). However, long-term application of these steroids results in detrimental side effects, including diabetes, immunosuppression, osteoporosis and increased risk of cardiovascular events, all of which are closely associated with the alteration of physiological metabolism by GCs (57). Thus, new anti-inflammatory drugs are urgently needed, ideally ones that maintain the effectiveness of GCs while avoiding the GC-associated detrimental effects. Bypassing GCs to activate GILZ represents a novel strategy to achieve anti-inflammation. In the current study, we found that short-chain alcohols (ethanol, propanol and isopropanol) upregulate GILZ without GCs, which deserves further investigation to explore if the alcohols can serve as prototype compounds to search for new anti-inflammatory agents.

Our previous genome-wide gene expression analysis on human airway epithelial cells that were exposed to dose-escalating ethanol (42) revealed that a cluster of glucocorticoid-targeting genes, including TSC22D3 (GILZ), ALOX15B, SYNPO2, and PTEN, responded in a dose-dependent manner. GILZ was the most affected, upregulated 2-fold by 50 mM ethanol and 3-fold by 100 mM ethanol (42). Importantly, GILZ is an essential molecule to convey the alcohol anti-inflammatory effect, as knockdown of GILZ diminishes alcohol suppression of LPS-induced inflammatory response (1). Our further research revealed that ethanol activation of the GILZ gene is through a non-canonical activation of the GR signaling pathway, which is independent of GCs (1). In the current study, we found that other similar short-chain alcohols (propanol and isopropanol) share the same property of modulating GILZ expression *in vitro* and *in vivo*, validating the novel alcohol-GR interaction. It is well studied that ethanol modulates the immune function of T cells, monocytes, macrophages, dendritic cells and neutrophils (4, 58–60), and the specific effects depend on the pattern of ethanol exposure (acute or chronic) (61). Acute ethanol decreases TLR responses and attenuates pro-inflammatory cytokine production (62, 63). However, chronic ethanol exposure renders monocytes and macrophages more responsive to LPS stimulation. Mechanistic studies demonstrate that the ethanol-induced LPS tolerance or sensitization is mediated through modulation of IRAK-M, IRAK1/4, Bcl-3, and NF-κB (14, 47). In contrast, the isopropanol-induced effect is conveyed through the regulation of discrete members of the NFAT family of transcription factors and AP-1 family of transcription factors (18, 19). In the current study, we demonstrate that short-chain alcohols modulate GILZ gene expression and suppress IκB phosphorylation, which adds another layer of regulation to the known mechanisms. Even though GILZ is known to interact with the key inflammatory signaling mediators NF-κB and AP-1 (32, 64, 65), the finding that short-chain alcohols exploit this mechanism for inflammosuppression and immunosuppression is novel. A recent publication reported that ethanol and other short-chain alcohols inhibit NLRP3 inflammasome activation through protein tyrosine phosphatase stimulation (66). It is noteworthy that stimulation of a functional inflammasome requires two steps. The first step is priming during which activation of NF-κB is essential to induction of several components of the inflammasome. Our data have shown that short-chain alcohols suppress IκB phosphorylation, which inevitably undermines the priming and the downstream activation of the inflammasome. It will be very interesting to investigate whether alcohol-induced protein tyrosine phosphatase stimulation subdues phospho-IκB production in future studies.

Despite intensive research, sepsis continues to be a major health problem world-wide. The incidence of sepsis in the past two decades has annually increased by 9%, to reach 240 per 100,000 people in the US (67, 68). This rate of occurrence translates into ~750,000 cases and over 250,000 deaths each year (68). When septic shock develops, the mortality rate of patients is substantially increased. At present, there is no specific treatment for sepsis and septic shock. Clinical management basically focuses on infection containment and organ function support (69). Alcohol attenuation of LPS-induced septic shock, as we demonstrated in this report, may be employed as an emergency measure to save lives under the circumstance of no medical care available. With regard to potential use of alcohol for therapy, recent studies have proposed to use ethanol to treat traumatic brain injury in humans (51, 52). Acute ethanol gavage attenuates hemorrhage/resuscitation-induced injury (70). Thus, alcohol, the oldest drug in medicine, may find new applications, as long as the molecular mechanism for its action is clearly understood.

While this is related to but beyond the scope of the current research, alcohol activation of GR signaling and upregulation of GILZ expression may be important in explaining alcohol-associated psycho-behavioral problems. Alcohol is long known to be an emotion regulatory agent. Moderate intake relieves stresses and produces pleasure, while heavy drinking induces mood and psychological abnormalities, such as depression. GILZ over-expression is found to be associated with depression (71, 72). Our data clearly demonstrated that short-chain alcohols upregulate GILZ, which may serve as a critical mechanism for alcohol mood regulation and alcohol-precipitated depression.

There are several limitations associated with this research. First, only acute and one-dose application of alcohols was tested. Multiple doses may have a stronger potency in immunosuppression against LPS. Second, this study was to prove the principle. Alcohols were applied prior to LPS challenge. However, in a clinical setting LPS septic shock typically occurs first. Future studies will test the clinically relevant mode. Third, in this report alcohols were administered intraperitoneally. Alternatively, gavage through a feeding tube is another clinically applicable way to deliver alcohols, which will be tested in the future.

In summary, this report demonstrated that short-chain alcohols upregulate GILZ expression, suppress host immune response to LPS, and attenuate LPS-triggered septic shock. This finding implies that short-chain alcohols can be used to alleviate LPS sepsis as an emergency measure if no other medicines are available.

## Data Availability Statement

All datasets generated for this study are included in the article/Supplementary Material.

## Ethics Statement

The animal study was reviewed and approved by the Institutional Animal Care and Use Committee of Louisiana State University Health Sciences Center with IACUC #3578.

## Author Contributions

HN, YW, and SJ performed experiments and data analyses. SN contributed to the original concept and design of the work. GW designed and conducted experiments, performed data analyses, and did manuscript writing.

### Conflict of Interest

The authors declare that the research was conducted in the absence of any commercial or financial relationships that could be construed as a potential conflict of interest.
