# Corrigendum: Short-Chain Alcohols Upregulate GILZ Gene Expression and Attenuate LPS-Induced Septic Immune Response

**DOI:** 10.3389/fimmu.2022.876794

**Published:** 2022-03-30

**Authors:** Hang Pong Ng, Yubo Wang, Scott Jennings, Steve Nelson, Guoshun Wang

**Affiliations:** ^1^ Department of Microbiology, Immunology and Parasitology, Louisiana State University Health Sciences Center, New Orleans, LA, United States; ^2^ Department of Medicine, Louisiana State University Health Sciences Center, New Orleans, LA, United States

**Keywords:** ethanol, propanol, isopropanol, anti-inflammation, immunosuppression, GILZ, LPS, septic shock

Yubo Wang was not included as an author in the published article. The corrected Author Contributions Statement appears below.

The authors apologize for this error and state that this does not change the scientific conclusions of the article in any way. The original article has been updated.

## Author Contributions

HN, YW and SJ performed experiments and data analyses. SN contributed to the original concept and design of the work. GW designed and conducted experiments, performed data analyses, and did manuscript writing.

## Publisher’s Note

All claims expressed in this article are solely those of the authors and do not necessarily represent those of their affiliated organizations, or those of the publisher, the editors and the reviewers. Any product that may be evaluated in this article, or claim that may be made by its manufacturer, is not guaranteed or endorsed by the publisher.

