# Supplementary material for: Short-Chain Alcohols Upregulate GILZ Gene Expression and Attenuate LPS-Induced Septic Immune Response
Source: Front Immunol. 2020 Feb 3;11:53. doi: 10.3389/fimmu.2020.00053 (PMC7008712; doi:10.3389/fimmu.2020.00053)
Supplement: Supplementary file 1 [file Data_Sheet_1.docx]

## SUPPLEMENTARY DATA

### Short-chain Alcohols Upregulate GILZ Gene Expression and Attenuate LPS-induced Septic Immune Response

Hang Pong Ng, Scott Jennings, Steve Nelson, Guoshun Wang

1. **Gating strategy to evaluate GILZ expression levels**


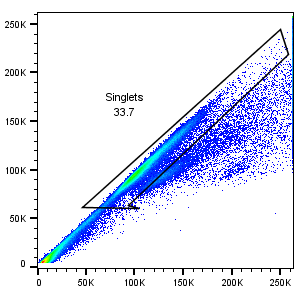

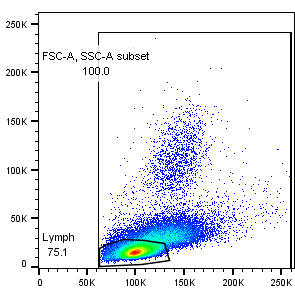

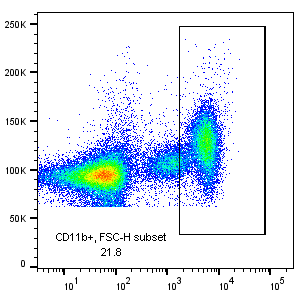

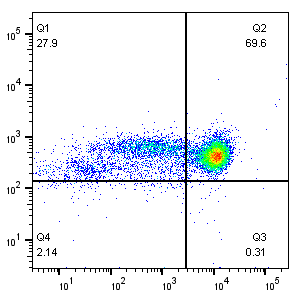

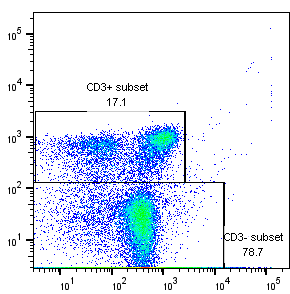

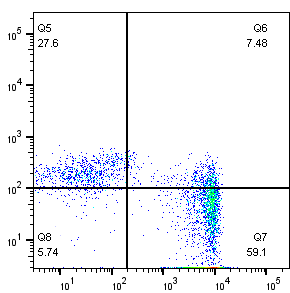

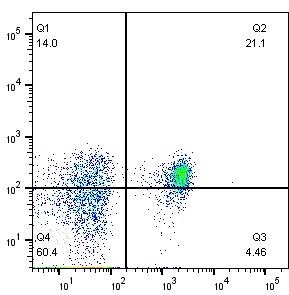

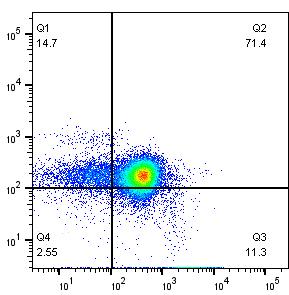


**FSC**

**-**

**A**

**FSC**

**-**

**H**

**Small Gate (Lymph subset)**

**Large Gate (FSC**

**-**

**A, SSC**

**-**

**A Subset)**

**PerCP**

**-**

**Cy5.5 :: CD19**

**Comp**

**-**

**FITC**

**-**

**A :: CD11b**

**Comp**

**-**

**APC**

**-**

**A :: Ly6G**

**FSC**

**-**

**A**

**SSC**

**-**

**A**

**Comp**

**-**

**Alexa**

**-**

**700**

**-**

**A::CD3**

**Comp**

**-**

**PE**

**-**

**Cy5**

**-**

**A :: CD4**

**FSC**

**-**

**A**

**Comp**

**-**

**Pacific Blue**

**-**

**A :: CD8**

**Upper Gate (CD3+ Subset)**

**Lower Gate (CD3**

**-**

**Subset)**

**Comp**

**-**

**PE**

**-**

**A :: GILZ**

**Comp**

**-**

**PE**

**-**

**A :: GILZ**

**Comp**

**-**

**PE**

**-**

**A :: GILZ**

**Comp**

**-**

**PE**

**-**

**A :: GILZ**

**FSC**

**-**

**A**

**FSC**

**-**

**H**

**Fig. S1. Gating Strategy to Evaluate GILZ Expression.** Nucleated cells from peripheral blood were blocked with TruStain FcTMXPLUS (2.5 µg/ml; BioLegend), and subjected to immunostaining with the following antibodies: CD11b-FITC (5 µg/ml; Invitrogen), Ly6G-APC (4 µg/ml; BD Pharmingen), CD3e-Alexa 700 (10 µg/ml; BD Pharmingen), CD8-Pacific Blue (5 µg/ml; BioLegend), CD4-PE-Cy5 (10 µg/ml; BD Pharmingen), and CD19-PerCP Cy5.5 (10 µg/ml; BD Pharmingen), Next, the cells were permeabilized and fixed using BD

Cytofix/Cytoperm™ Fixation/Permeabilization Kit. Then, the cells were intracellularly stained with GILZ-PE antibody (5 µg/ml; Invitrogen), followed by flow cytometry analysis.

1. **Gating strategy to evaluate phospho-IκB levels**


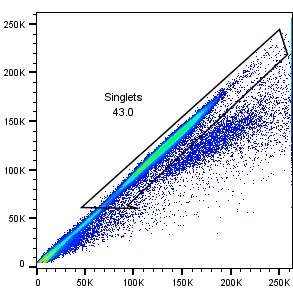

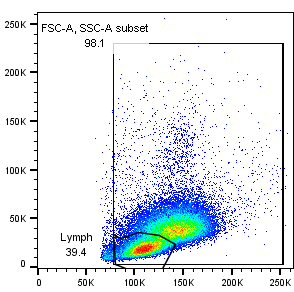

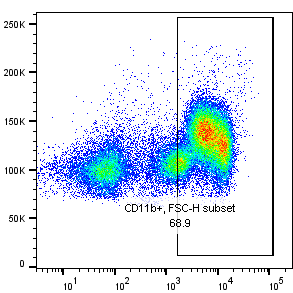

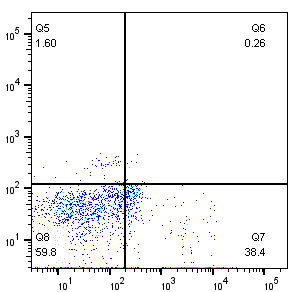

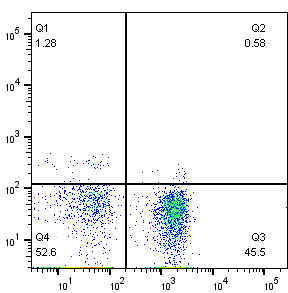

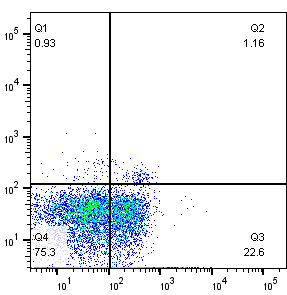

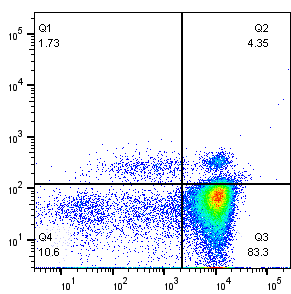


**FSC**

**-**

**A**

**FSC**

**-**

**H**

**Small Gate (Lymph subset)**

**Large Gate (FSC**

**-**

**A, SSC**

**-**

**A Subset)**

**PerCP**

**-**

**Cy5.5 :: CD19**

**Comp**

**-**

**FITC**

**-**

**A :: CD11b**

**Comp**

**-**

**APC**

**-**

**A :: Ly6G**

**Comp**

**-**

**PE**

**-**

**A ::**

**Phospho**

**-**

**I**

**κ**

**B**

**FSC**

**-**

**A**

**SSC**

**-**

**A**

**Comp**

**-**

**Alexa 700**

**-**

**A::CD3**

**Comp**

**-**

**PE**

**-**

**Cy5**

**-**

**A :: CD4**

**FSC**

**-**

**A**

**Comp**

**-**

**PE**

**-**

**A ::**

**Phospho**

**-**

**I**

**κ**

**B**

**Comp**

**-**

**Pacific Blue**

**-**

**A :: CD8**

**Comp**

**-**

**PE**

**-**

**A ::**

**Phospho**

**-**

**I**

**κ**

**B**

**Comp**

**-**

**PE**

**-**

**A ::**

**Phospho**

**-**

**I**

**κ**

**B**

**Upper Gate (CD3+ Subset)**

**Lower Gate (CD3**

**-**

**Subset)**


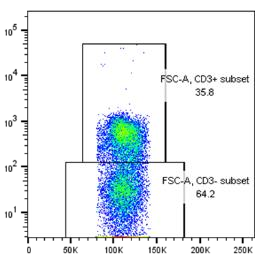


**FSC**

**-**

**H**

**Fig. S2. Gating Strategy to Evaluate Phosphor-IκB Levels.** Nucleated cells from peripheral blood were blocked with TruStain FcTMXPLUS (2.5 µg/ml; BioLegend), and subjected to immunostaining with the following antibodies against surface markers: CD11b-FITC (5 µg/ml; Invitrogen), Ly6G-APC (4 µg/ml; BD Pharmingen), CD3e-PE-Cy7 (10 µg/ml; BD Pharmingen), CD8-Pacific Blue (5 µg/ml; BioLegend), CD4-Alexa-700 (10 µg/ml; BD Pharmingen), and CD19-PerCP Cy5.5 (10 µg/ml; BD Pharmingen). Next, the cells were permeabilized and fixed using BD Cytofix/Cytoperm™ Fixation/Permeabilization Kit. Then, the cells were intracellularly stained with phospho-IκB-PE antibody (1.25 µg/ml; Invitrogen), followed by flow cytometry analysis.
